# Neurophysiological signatures of ageing: compensatory and compromised neural mechanisms

**DOI:** 10.1093/braincomms/fcaf131

**Published:** 2025-04-04

**Authors:** Kamalini G Ranasinghe, Kiwamu Kudo, Kaitlin Casaletto, Julio C Rojas-Martinez, Faatimah Syed, Keith Vossel, Bruce L Miller, Gil D Rabinovici, Joel H Kramer, Katherine P Rankin, Srikantan S Nagarajan

**Affiliations:** Department of Neurology, Memory and Aging Center, University of California San Francisco, San Francisco, CA 94158, USA; Department of Radiology and Biomedical Imaging, University of California San Francisco, San Francisco, CA 94143, USA; Medical Imaging Business Center, Ricoh Company, Ltd., Kanazawa 920-0177, Japan; Department of Neurology, Memory and Aging Center, University of California San Francisco, San Francisco, CA 94158, USA; Department of Neurology, Memory and Aging Center, University of California San Francisco, San Francisco, CA 94158, USA; Department of Neurology, Memory and Aging Center, University of California San Francisco, San Francisco, CA 94158, USA; Department of Neurology, David Geffen School of Medicine, Mary S. Easton Center for Alzheimer’s Disease Research, University of California Los Angeles, Los Angeles, CA 90095, USA; Department of Neurology, Memory and Aging Center, University of California San Francisco, San Francisco, CA 94158, USA; Department of Neurology, Memory and Aging Center, University of California San Francisco, San Francisco, CA 94158, USA; Department of Radiology and Biomedical Imaging, University of California San Francisco, San Francisco, CA 94143, USA; Department of Neurology, Memory and Aging Center, University of California San Francisco, San Francisco, CA 94158, USA; Department of Neurology, Memory and Aging Center, University of California San Francisco, San Francisco, CA 94158, USA; Department of Radiology and Biomedical Imaging, University of California San Francisco, San Francisco, CA 94143, USA

**Keywords:** healthy ageing, magnetoencephalography, neural oscillations, aperiodic slope, compromised and compensatory brain changes

## Abstract

Spatiotemporal patterns of neural oscillations change with ageing, even in the cognitively unimpaired individual. Whether these neurophysiological changes represent ageing-related vulnerabilities or mechanisms that support cognitive resilience remains largely unknown. In this study, we used magnetoencephalography imaging to examine age-related changes of resting-state whole-brain neurophysiology in a well-characterized cohort of cognitively unimpaired individuals (*n* = 70; age range 52–87 years). We quantified spatial patterns of age-related changes in band-limited spectral power within delta–theta (2–7 Hz), alpha (8–12 Hz) and beta (13–30 Hz) bands and the spectral aperiodic slope (15–50 Hz), and examined how spectral changes are associated with cognitive abilities in healthy ageing. In a subset of individuals (*n* = 40) who were evaluated with a uniform battery of cognitive tests, using a partial least square regression approach, we examined the associations between age-related spectral changes and cognitive performance. We found that, with advancing age, delta–theta and beta spectral power reduces, while alpha spectral power increases. A periodic slope also showed reductions with ageing. Better cognitive scores were positively correlated with delta–theta reductions and alpha power increases associated with ageing, suggesting that these may represent compensatory neural mechanisms. Beta power reductions and spectral aperiodic slope changes, in contrast, correlated negatively with higher cognitive scores, suggesting that these may represent compromised neural mechanisms of ageing. Our findings highlighted that the neurophysiological changes that occur during later decades of life were distinct from the previously known lifespan changes. This study demonstrates the trajectories of neurophysiological changes in cognitive ageing explicitly relating to conserved and impaired neural mechanisms with important implications for identifying specific spectral changes in neurodegenerative processes in the context of ageing.

## Teaser

Ranasinghe *et al*. reported changes in neural activity patterns of the brain in healthy older individuals ranging from 50 to 90 years. The authors identify specific changes that are compensatory and associated with better cognitive performance as well as those that are compromised and associated with poor cognitive performance.

## Graphical Abstract

**Graphical Abstract:**
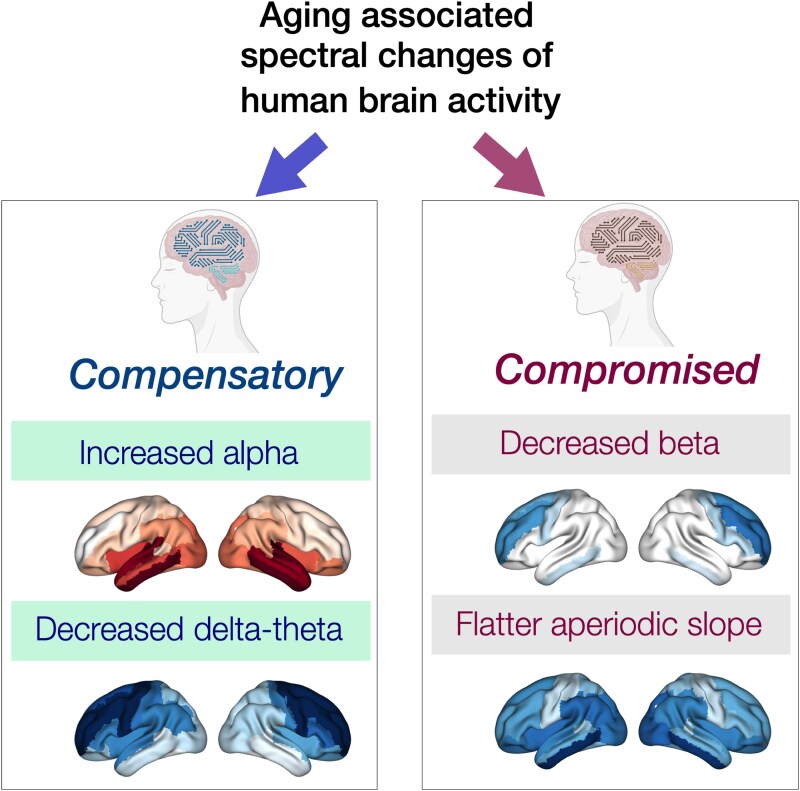

## Introduction

Cognitive changes in late life with intact ability to conduct everyday activities are considered normative. These changes may represent the physiological vulnerabilities of neural mechanisms to ageing as well as the compensatory mechanisms that subserve resilience.^1^ Both compromised and compensatory neural mechanisms reflect the collective activity of excitatory and inhibitory neural functions and can be captured in the neurophysiological spectral assays.^2^ Distinct neurophysiological patterns have indeed been described in different brain sates of young versus old, as well as in different brain traits such as cognitive ageing versus Alzheimer’s disease. For example, a gradual strengthening of alpha (8–12 Hz) spectrum is a well-documented change from infant to child in the human brain,^3^ while an increased delta–theta (2–7 Hz) with a reduced alpha (8–12 Hz) spectrum has been identified as the signature change in Alzheimer’s disease.^4^ Detailed neurophysiological spectral changes in ageing during the late decades of life and their relationships to cognitive ageing, however, remain incompletely understood.

Neurophysiological processes are represented in the power spectrum as two main components—narrowband oscillatory peaks representing the periodic activity and an exponential decrease of power across the frequency axis representing the aperiodic activity. The oscillatory periodic activity is frequently studied as band-limited spectral patterns, and the most salient of such in the adult resting human brain is an oscillating peak in the alpha band (8–12 Hz).^5^ The lower-frequency spectrum of delta–theta (2–7 Hz) and the higher beta (13–30 Hz) is also conventionally quantified for oscillatory power, although these peaks are less consistent in the resting human brain. Population studies including individuals across the lifespan have shown a drop in resting-state alpha oscillatory activity in the fourth decade compared with second and third.^6^ However, a closer look at alpha changes in the ages 50 years and above shows mixed findings of increased as well as decreased alpha power in the resting brain.^6-11^ Previous studies have also shown that alpha peak frequency—the frequency within the alpha power is highest, shows a reduction in the old when compared with the young.^12,13^ Most studies that examined the lifespan changes of delta–theta and beta and have reported decreased delta–theta during developmental years and relatively low beta activity in early childhood.^7^ Other studies have also reported changes in spectral power across the full lifespan, indicating a general trend of reduced power in the low-frequency oscillations.^14,15^ In addition to these studies focused on the periodic oscillatory activity, the spectral aperiodic slope has come to sharp focus recently as an indicator of excitatory–inhibitory balance in local neural networks.^16-18^ A simple computational model delineated the specific associations of spectral aperiodic slope where greater excitability is indicated by a reduced (flattened) slope.^19^ Flatter slopes have indeed been consistently reported in older compared to young^17,18,20^ indicating ageing-related neuronal hyperexcitability. While these studies have clearly demonstrated that both periodic and aperiodic features change from young to old, they do not elaborate on the distinct changes that take place within the specific window of old age where progressive changes may occur in the latter decades of life. Detailed temporal evolution of spectral changes in the ageing brain in healthy elderly individuals and how these changes may relate to cognitive abilities still remain incompletely understood. Such knowledge, however, is key not only to inform strategies to improve brain health in ageing but also to help develop therapies to improve cognition in neurodegenerative diseases such as Alzheimer’s disease.

The current study was designed to understand detailed spectral patterns of neurophysiological changes in healthy ageing and how these relate to cognitive abilities. We leveraged high spatiotemporal resolution of magnetoencephalography (MEG) to quantify neural power spectrum in cognitively unimpaired adults (55–90 years; *n* = 70). Band-limited spectral power was estimated within three frequency bands: delta–theta (2–7 Hz), alpha (8–12 Hz) and beta (13–30 Hz), and spectral aperiodic slope was computed for 15–50 Hz frequency range. Our goal was to characterize the normative trajectories of regional neurophysiological signatures in cognitively unimpaired elderly between 55 and 90 years. We then identified compensatory and compromised neurophysiological manifestations in healthy ageing, based on the associations between spectral patterns and cognitive scores in executive, memory and processing speed abilities. Specifically, using a partial least square regression (PLSR) approach, we identified compensatory neurophysiological changes that are positively associated with higher cognitive scores and compromised changes that are negatively associated with higher cognitive scores. This study lays the foundation to better understand neurophysiological abnormalities that occur in dementia syndromes in the context of healthy ageing.

## Materials and methods

### Participants

In this cross-sectional study, we included 70 cognitively unimpaired older adults who were evaluated at the UCSF Memory and Aging Center (UCSF-MAC). Participants represented a community-dwelling sample of older adults in the San Francisco Bay Area. All participants underwent clinical assessment as operationalized by the Clinical Dementia Rating (CDR)^21^ via study partner interviews and demonstrated either no (97% CDR = 0) or minimal (3% CDR = 0.5) functional deficits (Table 1). A subset of 40 participants were evaluated with a uniform cognitive battery to examine executive, memory, and processing speed performances (see Supplementary methods for details). The sub-cohort of *n* = 40 subjects (62.5% female) were not different in their demographic and cognitive measures (Supplementary Table 1) from the full cohort of 70 subjects (60% female). A structural MRI of the brain was also collected from each participant to be used in the source-space reconstruction of MEG signal processing. Informed consent was obtained from each participant, and the study was approved by the UCSF Institutional Review Board.

**Table 1:** Demographic characteristics and cognitive test performance of participants

Variable	50–64 years (*n* = 21)	65–74 years (*n* = 29)	75–90 years (*n* = 20)	*P*-value^a^
Age (years)	60.05 ± 3.79	70.18 ± 2.97	79.48 ± 3.54	-
Sex (% female)	12 (57%)	18 (62%)	12 (60%)	0.9403
Handedness (% right)	17 (81%)	20 (72%)	17 (85%)	0.5391
Race (% white)^b^	18 (95%)	24 (83%)	18 (100%)	0.1574
Education (years)	17.45 ± 1.61	17.34 ± 1.95	17.55 ± 2.37	0.9386
MMSE	29.71 ± 0.56	29.38 ± 1.08	29.20 ± 0.89	0.1824
CDR	0.02 ± 0.11	0.02 ± 0.09	0.0 ± 0.0	0.6485
CDR-SOB	0.05 ± 0.15	0.02 ± 0.09	0.03 ± 0.11	0.6606
Modified trails	0.76 ± 0.25	0.59 ± 0.25	0.62 ± 0.24	0.1177
Design fluency	13.43 ± 3.27	12.43 ± 4.01	12.83 ± 3.29	0.7204
Phonemic fluency	18.89 ± 2.93	18.00 ± 7.2	16.28 ± 3.58	0.4376
Category fluency	24.54 ± 5.88	23.88 ± 5.33	23.56 ± 3.50	0.8608
Digit span forwards	7.71 ± 1.54	7.13 ± 1.36	6.78 ± 1.06	0.1463
Digit span backwards	5.57 ± 1.02	5.91 ± 1.56	5.67 ± 1.24	0.7208
Processing speed	94.23 ± 9.10	89.05 ± 17.8	78.44 ± 12.78	**0**.**0105**^c^
Stroop inhibition	60.46 ± 9.77	53.05 ± 14.2	44.83 ± 13.37	**0**.**0065**^c^
Modified Rey copy	15.89 ± 0.60	15.33 ± 0.86	15.5 ± 0.71	0.1980
VOSP number location	9.54 ± 0.78	9.13 ± 1.36	8.83 ± 1.29	0.2941
Repetition	4.92 ± 0.28	4.87 ± 0.34	4.72 ± 0.57	0.3764
CVLT learning	56.74 ± 9.61	50.74 ± 10.88	52.65 ± 10.9	0.1697
CVLT short-delay recall	12.42 ± 2.76	11.67 ± 2.95	11.80 ± 2.93	0.6676
CVLT long-delay recall	13.21 ± 2.72	11.81 ± 3.03	12.60 ± 2.80	0.2659
Modified Rey recall	13.3 ± 3.16	11.29 ± 3.08	12.44 ± 1.92	0.1414
GDS	2.69 ± 2.81	2.82 ± 3.03	2.29 ± 1.72	0.8196

Values for all variables except for sex, handedness, and race are means ± SD. Bold values indicate the tests that showed statistical significance in groupwise analyses.

^a^ *P*-values are reported from one-way ANOVA with pairwise comparisons for age, education, and all the cognitive tests and from Pearson χ^2^ test for sex, race and handedness.

^b^Race was self-reported. Two participants in 50–64 age group and two participants in 75–90 age group opted out from reporting the race.

^c^Pairwise comparison showed statistically significant difference between 50–64 age group and 75–90 age group for processing speed (Stroop colour naming; *P* = 0.0114) and Stroop inhibition tests (*P* = 0.0048).

CDR, Clinical Dementia Rating; CDR-SOB, CDR-Sum of Boxes; CVLT, California Verbal Learning Test (16-item version); GDS, Geriatric Depression Scale; MMSE, Mini Mental State Examination; VOSP, visual object and space perception battery.

### Resting-state MEG acquisition and analysis

Each subject underwent MEG on a whole-head biomagnetometer system consisting of 275 axial gradiometers (MISL, Coquitlam, BC, Canada). A minimum of 10 min of continuous data was collected from each subject while lying supine, awake, and eyes-closed (sampling rate: 600 Hz). The data collection was done under the same protocols described previously.^22^ Data were pre-processed using the Fieldtrip toolbox, and source-space reconstruction was performed using custom-built MATLAB tools.^23^

#### Band-limited spectral power

We computed the spectral power (relative spectral power) within delta–theta (2–7 Hz), alpha (8–12 Hz), and beta (13–35 Hz) bands, for 210 cortical regions defined in the Brainnetome atlas, for each participant. In our analysis, we examined the low-frequency activity as a combined delta–theta band ranging from 2 to 7 Hz. The rationale to combine this frequency activity into a single band is based on the fact that delta and theta band oscillatory activity does not show clear divergence of effects either with ageing or in neurological diseases.^8,24,25^ This lack of distinctive boundary between delta and theta may arise either from shared biology or from signal leakage of low-frequency filtering. Our approach to combine the delta and theta bands into a single band increases the sensitivity of the low-frequency spectrum. Because the most common conventional approach to examine the low-frequency component is to separate delta (2–4 Hz) and theta (4–8 Hz) bands, we ran our statistical models (spline and linear fits as described below), on these conventional band-limited power spectral density (PSD) as well (Supplementary Fig. 1). This analysis showed that isolated theta band activity was identical to the combined delta–theta band change with ageing, while isolated delta band activity did not show a specific generalized directional change−a finding which further supported the presumed arbitrary nature of delta and theta into two frequency components.

#### Peak frequency

We computed the peak frequency within 8–12 Hz band, for each cortical region.

#### Aperiodic slope

In each region, the aperiodic spectral component, $\mathrm{PS}D_{\mathrm{aper}}(f)∼1/f^{\alpha },$ was extracted using a three-step least square procedure,^26^ and the spectral exponent *α* was evaluated as the slope of the linear regression of spectral power. Previous studies have suggested that the aperiodic spectral slope of high frequency range is reflective of local neuronal excitability, where a flatter slope indicates greater neural excitability.^17,19^ Furthermore, previous studies have clearly demonstrated that the most reliable frequency spectrum to estimate aperiodic slope is the frequency range of 15–50 Hz.^19,27^

### Statistical analyses

#### Hierarchical mixed-effects models

We used mixed models in our full sample (*n* = 70), to examine the relationships between age and spectral power within the frequency bands of delta–theta, alpha and beta. The hierarchical repeated measures design included spectral power values per each cortical region (210 regions) nested into 40 modular levels (Brainnetome atlas). We used the MIXED procedure in SAS to fit a linear mixed model (LMM) and GLIMMIX procedure to fit a spline model. Specifically, the spline fits (GLIMMIX) were used to demonstrate a smooth approximation of all the datapoints (spectral changes in all brain regions across age), while the linear fits (MIXED) were used to determine the overall directional trajectory of change that can be used to infer conclusions based on statistical thresholds. Each frequency band was tested using a separate model. Both models (MIXED and GLIMMIX) included age as the predictor variable and the frequency-specific spectral power as the dependent variable. A separate set of LMMs were used where age was included as a categorical predictor. Specifically, we separated the full cohort (*n* = 70) into three tercile bins according to age: 50–64 years (*n* = 21), 65–74 years (*n* = 29) and 75–90 years (*n* = 20). We tested each cortical module (PROC MIXED) in a separate model [component regions of interest (ROIs) included as repeated measures]. Multiple comparisons were corrected at false discovery rate (FDR) 5%. The models examining the age-related changes of band-limited spectral power included aperiodic slope as a covariate. Identical LMMs were used to examine the associations between age and aperiodic slope including both MIXED and GLIMMIX procedures.

#### Comparison between below-median and above-median agers

To identify the associations between spectral measures and cognition, we first used a median split approach in the subset of *n* = 40 participants who were assessed with uniform cognitive measures within 7.8 ± 5.27 months of the MEG scan. Specifically, we identified the individuals who belonged to the upper median range in their performance in all three cognitive measures: memory-composite, executive-composite and processing speed. This analysis identified 10 individuals as ‘above-median’ group and the rest (*n* = 30) as ‘below-median’ group. Next, we used LMMs to compare frequency-specific spectral power and aperiodic slope between above-median subgroup and below-median subgroup. The models included age as a covariate, subject identity and ROIs as repeated measures. To examine the spatial patterns of changes between above- and below-median subgroups, we used similar hierarchical LMMs at each modular level (component ROIs included as repeated measures), including age as a covariate. Outputs were thresholded at 5% FDR.

#### Partial least square regression (PLSR)

We used PLSR analyses^28^ to examine the associations between age, cognition and frequency-specific spectral changes using the subset of participants (*n* = 40 cohort) who were assessed with a uniform battery of cognitive tests.^29^ Specifically, we used age-corrected summary *z*-scores for memory and executive function and the processing speed scores. Neural data for PLSR models included the spectral power metrics for alpha, delta–theta and beta bands and the aperiodic slope. This analysis was specifically geared to achieve a low dimensional output from a cognitive and neural correlation in order to derive meaningful conclusions about which frequency changes are associated with better cognitive performance in memory, executive and processing speed abilities in healthy ageing. Specifically, the PLSR allows correlating multidimensional data sets (collected from a single cohort) by creating latent variables which are obtained as linear combinations of the original variables and representing the maximal covariance. PLSR can also handle the case of multi-colinear predictors making it a very versatile tool to handle large data sets for which standard regression methods are not optimally suited.^28^ Our PLSR models were used as fixed-effects models as their purpose was to illustrate the relationship between neural and cognitive ageing data (not to generate a predictive model). Neural data were independent variables, and the three cognitive domain scores (memory, executive and processing speed) and age were dependent variables. Separate PLSR models were used for each band-specific spectral power and for aperiodic slope. In each case, we examined the neural and behavioural scores projected onto the latent space created by the two latent dimensions (LDs) (LD1 versus LD2) generated by PLSR.

## Results

Seventy (*n* = 70) elderly individuals without clinical evidence of cognitive impairment were included in the study (Table 1). The age range of the study cohort at the time of MEG evaluation was 52.54–87.13 years. We examined the demographic and cognitive performance across age, by categorizing the subjects into tercile bins according to age distribution of the sample: 50–64 years (*n* = 21), 65–74 years (*n* = 29) and 75–90 years (*n* = 20), which facilitated a meaningful demonstration of age-related changes of cognition. The three categorical groups were matched in the demographics of sex, education, race and handedness. Stroop colour naming and Stroop inhibition were the only neuropsychological tests that showed a decline with ageing, showing significant reductions in 75–90 years group compared with the 50–64 years group (Table 1).

### Age-related changes of band-limited spectral power and aperiodic slope

**Figure 1:**
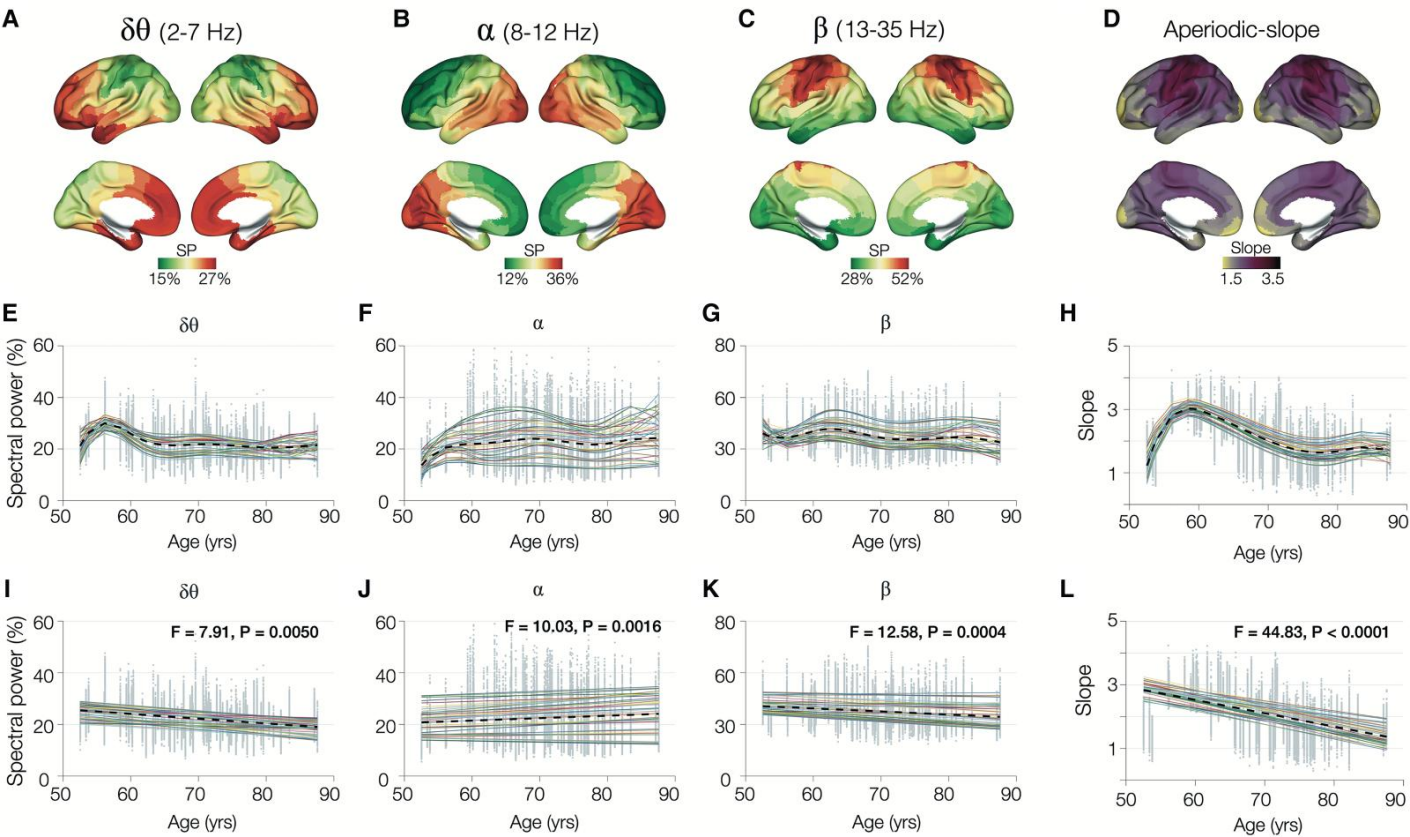
**Spectral changes with ageing.** Band-limited spectral power within delta–theta (2–7 Hz), alpha (8–12 Hz) and beta (13–30 Hz) frequency bands and spectral aperiodic slope within 15–50 Hz is examined in the full cohort (*n* = 70). The brain renderings display the average spectral power values for each frequency band. Delta–theta spectral power distribution showed a frontal predominant distribution (**A**), alpha showed an occipito-temporal predominant distribution (**B**) and beta showed a parieto-central predominant distribution (**C**). Exponent of the aperiodic spectral component showed reduced slopes in the frontal and temporal lobes (**D**). Spline fits for region-specific spectral data (Brainnetome atlas modules) showed the trajectory of change across age for each band-limited spectral power (**E–G**), and a linear model fit showed a low-grade increase of alpha power with ageing (**J**) but a low-grade decrease in delta–theta (**I**) and beta power with ageing (**K**). Spline fits showed the trajectory of aperiodic slope change across age (**H**), and a linear model fit showed decreasing aperiodic slope with ageing (**L**). Coloured lines in subplots **E–L** represent the spectral measures in each of the 48 modular-level parcellations defined in the Brainnetome atlas, and dotted lines represent the average across all regions. SP, spectral power.

The regional patterns of band-limited spectral power within delta–theta, alpha and beta frequency bands showed unique distributions. Specifically, delta–theta showed a frontal–temporal predominant distribution (Fig. 1A), alpha showed the classic occipital predominant distribution (Fig. 1B), while beta showed a central predominant distribution (Fig. 1C). The average spatial distribution of aperiodic slope in cognitively unimpaired elderly showed reduced slope in frontal and temporal regions (Fig. 1D). In this analysis, we computed the exponent of the spectral aperiodic component and depicted this measure as positive values (sign-inverted negative slopes), where lower values indicate flatter slopes.

Using spline and linear model fits, we next examined the age-associated changes in band-limited spectral power and aperiodic slope. The spline fits across ages for delta–theta power showed an initial window of increase during 52–58 age range which then dropped to the initial levels by age 65 years and followed a stable pattern until 80–90 years (Fig. 1E). The spline fits for alpha spectral power showed an initial increase during the 55–60 age range which then stayed stable at this elevated range until 80–90 years (Fig. 1F). The spline fits of beta spectral power showed a stable pattern throughout the ages of 55–90 years (Fig. 1G). In linear model fits, both delta–theta and beta showed slightly negative slopes, illustrating an age-related gradual reduction of spectral power (Fig. 1I and K; delta–theta: β = −0.186, *P* < 0.0001; beta: β = −0.177, *P* < 0.0001), while alpha showed a slightly positive slope, indicating an age-related gradual increase of spectral power (Fig. 1J; alpha: β = 0.095, *P* < 0.0001). With regard to spectral aperiodic slope, the spline fits across ages showed an initial increase during 52–60 age range followed by a steady drop until around 80 (Fig. 1H). A linear model fit showed reduced aperiodic slope associated with increasing age, indicating greater degree of hyperexcitability with ageing (Fig. 1L; aperiodic slope: β = −0.042, *P* < 0.0001). We also examined the effect of sex on spectral changes in ageing. LMMs predicting each frequency-specific spectral PSD showed that sex does not have any significant main effects (delta–theta: *F* = 0.001, *P* = 0.98; alpha: *F* = 0.01, *P* = 0.94; beta: *F* = 1.60, *P* = 0.21) or interaction effect with age (delta–theta: *F* = 0.01, *P* = 0.92; alpha: *F* = 0.03, *P* = 0.86; beta: *F* = 1.93, *P* = 0.16). Similar LMMs predicting spectral aperiodic slope showed a significant effect of sex where females showed a greater reduction than males (main effect: *F* = 4.73, *P* = 0.029; interaction: *F* = 4.63, *P* = 0.015).

**Figure 2:**
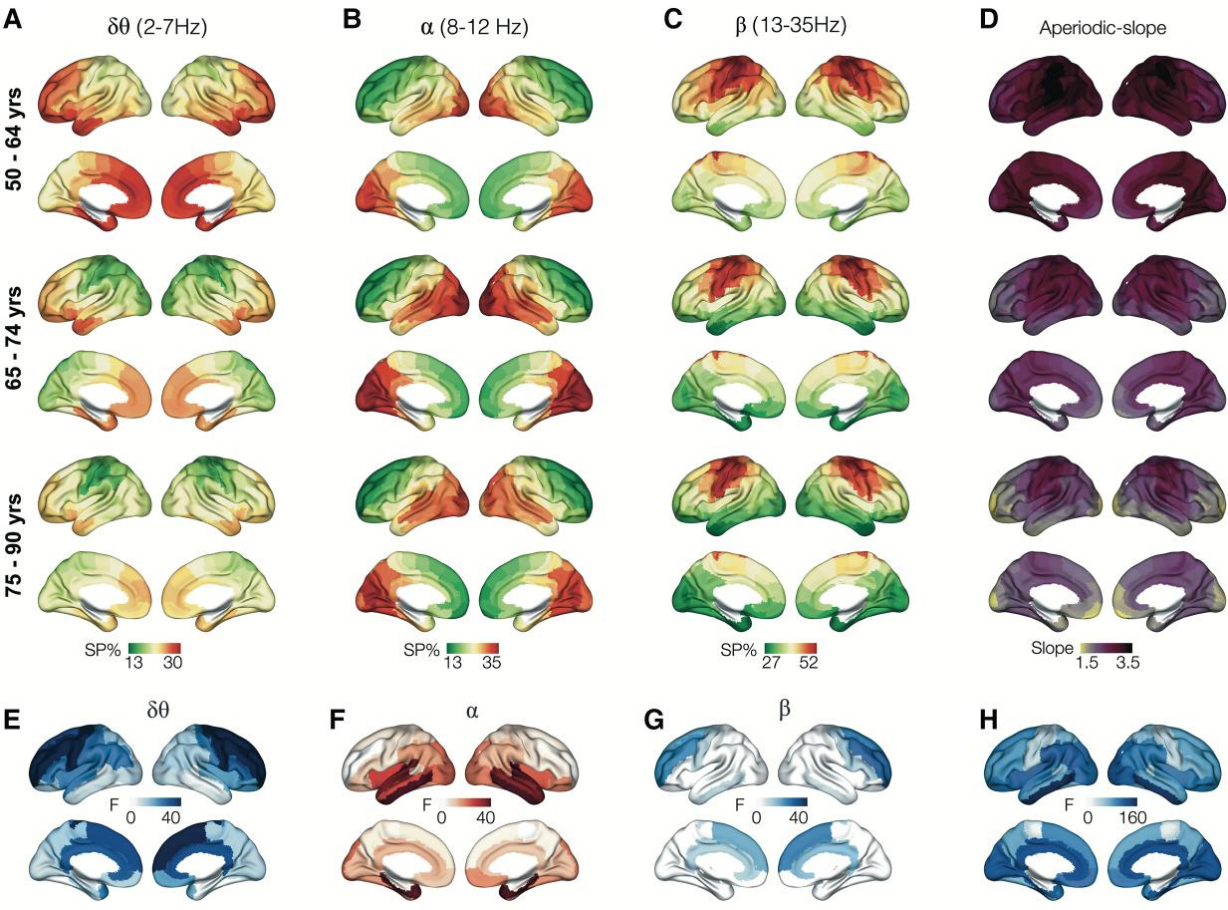
**Spatial patterns of band-limited spectral power and aperiodic slope changes with ageing.** Band-limited spectral power is examined within delta–theta (2–7 Hz), alpha (8–12 Hz) and beta (13–30 Hz) frequency bands and within each tercile bin of the age distribution in the full cohort, which included the age bins of 50–64 years (*n* = 21), 65–74 years (*n* = 29) and 75–90 years (*n* = 20). The brain renderings depict the average spectral power as a percentage (%) in a regional analysis for the cortical regions defined in the Brainnetome atlas in each cohort. When considering the pattern of age across the three tercile bins, delta–theta showed a reduction of spectral power (**A**), alpha showed a slight increase in spectral power, especially from 50–64 range to 65–74 range (**B**), while beta showed a reduction of spectral power (**C**). The colour bars are scaled from minimal to maximal spectral power % values within each frequency band as shown. Spectral aperiodic slope showed a reduction from 50–65 to 75–90, especially over the frontal and inferolateral parietal regions (**D**). An LMM analysis examining the changes across the three tercile bins, within band-limited spectral power for delta–theta (2–7 Hz), alpha (8–12 Hz) and beta (13–30 Hz), showed region dependency of these changes, where delta–theta reductions are predominantly frontal (**E**), alpha increases are predominantly temporal (**F**) and beta reductions are predominantly frontal (**G**). Spectral aperiodic slope changes were widely distributed with inferolateral temporal and cingulate cortices showing the greatest reductions with age (**H**). The images in **E–H** show significant regions after thresholded at FDR 5%. SP, spectral power.

Next, we used an LMM approach, across the three tercile bins depicting 50–64 years, to 65–74 years to 75–90 years, to examine the spatial patterns of age-related spectral changes (Supplementary Fig. 2A). The reduction of band-limited spectral power in both delta–theta and beta was most predominant in the frontal regions of the brain (Fig. 2A, C, E and G), while the spectral power increase within alpha band was most predominant in medial and lateral temporal cortices (Fig. 2B and F). These models also included aperiodic slope as a covariate to regress out any effect of aperiodic activity in the comparisons. An LMM analysis examining the aperiodic slope reductions across the three tercile bins of ageing showed widespread reductions of slope with predominant involvement of medial and lateral temporal lobes and cingulate cortex (Fig. 2H). We also examined the spatial patterns of peak frequency within the 8–12-Hz frequency band across the three tercile bins. This analysis demonstrated the posterior predominant distribution of alpha oscillatory activity and showed that spatial pattern of peak frequency remains stable with ageing (Supplementary Fig. 2B).

### Associations of ageing-related spectral changes and cognitive abilities: a median split analysis

**Figure 3:**
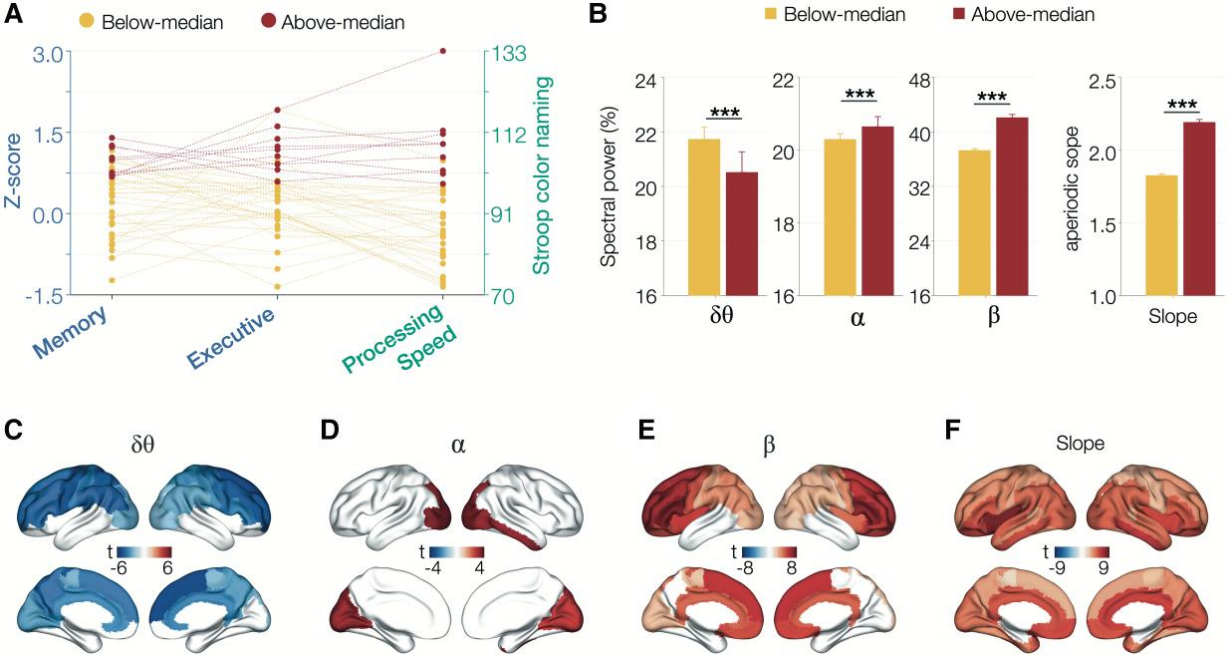
**Associations between spectral changes and cognitive performance in healthy ageing: a median split analysis.** A median split analysis identified above-median subgroup (*n* = 10) and below-median subgroup (*n* = 30) based on the performance on memory, executive and processing speed functions (**A**). This analysis included the subset of individuals (*n* = 40) who underwent uniform cognitive assessments memory, executive and processing speed functions. A ROI-based LMM analysis that examined the specific spectral measure between the two groups from median split. For band-limited frequency measures, above-median subgroup has lower delta–theta (*t* = −12,0, *P* < 0.0001), higher alpha (*t* = 3.91, *P* < 0.0001) and higher beta (*t* = 15.93, *P* < 0.0001) spectral power compared with the below-median subgroup (**B**). A similar LMM showed that above-median subgroup has higher aperiodic slopes (*t* = 24.70, *P* < 0.0001) compared with below-median agers (**B**). An LMM analysis examining the spatial patterns of changes between above- and below-median subgroups, within band-limited spectral power for delta–theta (2–7 Hz), alpha (8–12 Hz) and beta (13–30 Hz) and aperiodic slope, showed region dependency of these changes, where delta–theta reductions were frontally distributed (**C**), alpha increases were temporo-occipital (**D**) and beta reductions were widespread but most predominant in the frontal lobes (**E**). Aperiodic slope increases in above-median subgroup was widely distributed involving all regions of the cortex (**F**). The images show significant regions in the group contrast analysis between below-median (*n* = 30) and above-median (*n* = 10) subgroups and after thresholding at FDR 5%.

Next, we examined the associations between the specific spectral changes with ageing and cognitive abilities in our elderly cohort. This analysis included a subset of 40 (*n* = 40) subjects who were evaluated with a uniform battery of standard neuropsychological tests belonging to three main cognitive domains: memory, executive and processing speed. First, using a median split in the domain-specific composite cognitive scores, we identified individuals who belonged to the above-median subgroup (*n* = 10) and below-median subgroup (*n* = 30) in our sample (see methods for details; Fig. 3A). A linear mixed-effects model including age as a covariate showed that above-median subgroup have significantly lower delta–theta power (Fig. 3B; *t* = −12.00, *P* < 0.0001, CI: 20.29–20.77 and 22.11–22.39, for above- and below-median subgroups, respectively) and higher alpha power (Fig. 3B; *t* = 3.91, *P* < 0.0001, CI: 21.15–21.84 and 20.49–20.88, for above- and below-median subgroups, respectively) compared with below-median subgroup. The spatial patterns of spectral changes between above- and below-median subgroups, using linear mixed-effects models, demonstrated frontal predominant spatial distribution of delta–theta reductions and temporo-occipital predominant spatial distribution of alpha increases in the above-median subgroup compared with the below-median subgroup (Fig. 3C and D). In delta–theta and alpha, both the direction of power change and the regional patterns observed in the above-median subgroup were identical to spectral changes of ageing observed in these bands (Figs 1 and 2), indicating that these changes likely represent compensatory neural activity patterns supporting better cognitive health in ageing.

Compared with the below-median subgroup, the above-median subgroup showed higher beta power and greater aperiodic slopes (Fig. 3B; beta power: *t* = 15.93, *P* < 0.0001, CI: 37.23–37.91 and 34.12–34.50, for above- and below-median subgroups, respectively; aperiodic slope: *t* = 24.70, *P* < 0.0001, CI: 2.17–2.22 and 1.81–1.84, for above- and below-median subgroups, respectively). These changes in the above-median subgroup sharply opposed what was observed with ageing, which showed reduction of beta power and reduction of slope (Figs 1 and 2). As such, beta power and slope reductions in ageing may represent compromised neural activity patterns in the ageing brain. Specially, with respect to aperiodic slope, such compromised neural mechanisms may represent abnormal neural circuit hyperexcitability of ageing. The spatial patterns of increased beta power in the above-median subgroup involved the frontal (Fig. 3E). The spatial patterns of increased aperiodic slope in the above-median subgroup compared with the below-median subgroup were widely distributed involving the whole cortex, with the highest values in temporal and cingulate cortices (Fig. 3F), which were indeed the same regions which showed slope reductions in ageing (Fig. 2H). The spatial consistency may further allude to preserved neural excitability in the above-median subgroup associated with better cognitive scores.

### Associations of ageing-related spectral changes and cognitive abilities: a PLSR analysis

**Figure 4:**
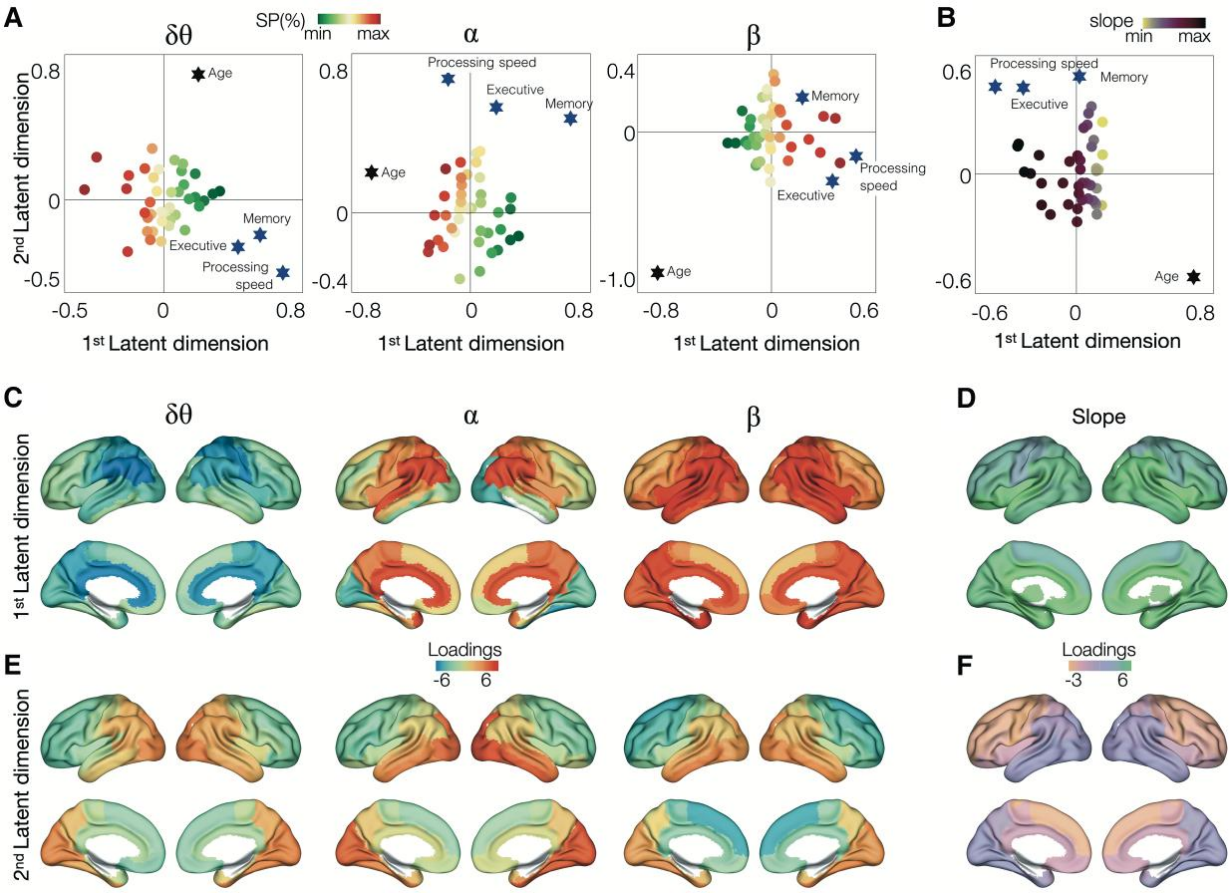
**Associations between spectral changes and cognitive performance in healthy ageing: a PLSR analysis**. A PLSR with two LDs combining the spectral changes and cognitive function in executive, memory and processing speed domains, as well as age, demonstrated the associations between neural and cognitive measures in the latent space depicted by LD1 versus LD2 for band-limited spectral power (**A**) and for aperiodic slope (**B**). In the three panels shown in subplot (**A**), each dot indicates a subject (*n* = 40) and the colour scale from green shades to red shades indicates low to high spectral power within each frequency band of delta–theta, alpha and beta, respectively, from left to right. Lower delta–theta spectral power and higher cognitive scores were associated with LD1 (left panel), higher alpha spectral power and higher cognitive scores were associated with LD2 (middle panel) and higher beta spectral power and higher cognitive scores were associated with LD1 (right panel), in each respective PLSR model. In subplot (**B**), each dot indicates a subject (*n* = 40) and the colour scale from light to dark indicates low to high aperiodic slope. Lower aperiodic slope values were associated with LD1, and higher cognitive scores were associated with LD2. Subplots (**C**)–(**F**) depict the regional patterns of factor loadings: first LD derived from PLSR of band-limited spectral power measures (**C**); first LD derived from PLSR of aperiodic slope (**D**); second LD derived from PLSR of band-limited spectral power measures (**E**); second LD derived from PLSR of aperiodic slope (**F**). SP, spectral power.

Next, we used a PLSR analysis with fixed-effects models using two latent factors to illustrate the multidimensional relationships between neural data, cognitive scores and age to examine the associations between spectral changes in ageing and domain-specific cognitive scores. Subplots 4A depict these associations plotted against the first and second LDs derived from PLSR analysis for each band-limited spectral power measures. The PLSR models displayed distinct neural behavioural associations along the first and second LDs. For example, the first LD of delta–theta PLSR model was positively associated with better cognition (cognitive scores were distributed at the positive end of LD1; Fig. 4A left panel), but negatively associated with spectral power (from red to green depict high to low along LD1), collectively illustrating that higher cognitive scores are associated with lower delta–theta power (Fig. 4A left panel). This is consistent with the findings from the median split analysis where above-median subgroup showed lower delta–theta power. Age also showed a relatively smaller (closer to zero) positive association with LD1 again showing consistency with the finding that delta–theta spectral power reducing with age. In the alpha band PLSR, higher cognitive scores as well as age showed a positive association with the second LD (Fig. 4A middle panel). Spectral power distribution of alpha showed a somewhat diagonal distribution in the LD1–LD2 space, where higher power seems to be associated with ageing as well as higher cognitive scores (Fig. 4A middle panel). This is again consistent with the findings from the median split analysis where above-median subgroup showed higher alpha power than below-median subgroup. In the PLSR model of the beta band, the first LD was positively associated with higher cognitive scores, as well as with higher spectral power (Fig. 4A right panel, red dots distributed along the positive side of the LD1). This finding is consistent with the earlier finding that above-median subgroup have higher beta power than below-median subgroup. The LD1 of beta power PLSR showed a strong negative association with age (Fig. 4A, right panel). Collectively, the PLSR analyses demonstrate that preserving higher power within alpha and lower power within delta–theta bands supports better executive and memory functions and faster processing speed abilities during the sixth–ninth decade of life.

We also examined the spatial patterns of the LDs along which the cognitive scores are correlated with band-limited spectral power. For example, frontal and temporal regions contributed highest to the first LD of delta–theta PLSR (Fig. 4C left panel). The second LD of delta–theta PLSR (Fig. 4E left panel) was contributed highest by occipital cortices, although this dimension showed relatively weak association with cognitive sores. For the alpha band, it was the second LD which showed stronger relationship with the distribution of cognitive scores, and we found that inferolateral temporal cortex and parieto-occipital regions contribute mostly to the second LD (Fig. 4E middle panel). Both delta–theta and alpha spectral changes with ageing were in the same direction with the changes associated with better cognitive abilities, suggesting that delta–theta reductions in the frontal cortices and alpha increases in the inferolateral temporal and parieto-occipital cortices may represent compensatory neural mechanisms that support better cognitive outcomes in ageing. LD1 of beta band PLSR, which was the dimension associated with the distribution of cognitive scores, was mostly contributed by higher beta power within lateral temporal and parieto-occipital regions, indicating their positive regional relationship to better cognitive scores (Fig. 4C right panel). However, as shown in Figs 1 and 2, beta spectral power reduces with ageing, hence signalling that beta changes of ageing are not supporting better cognitive status, suggesting that beta power reductions may reflect ageing-related impairments in neural activity.

A similar PLSR model for the aperiodic slope illustrated that higher cognitive scores are positively associated with the second LD, whereas age was negatively associated with LD2 and positively associated with LD1 (Fig. 4B). Consistent with the ageing-associated reductions, aperiodic slope values were clearly distributed from high to low (from dark to light) along the LD1 (Fig. 4B). Aperiodic slope did not show a clear distribution from high to low along the second LD, suggesting a weak association with cognitive scores, a result less consistent with the median split finding. The regional patterns of LD2 showed that inferolateral temporal cortex and parieto-occipital regions contributed mostly towards this dimension, indicating that higher slope values in these regions are associated with better cognitive scores which are positively correlated with LD2 (Fig. 4D and F). Collectively, the direction of aperiodic spectral change was opposite to the direction of change associated with higher cognitive scores, suggesting that aperiodic slope reductions depicting network hyperexcitability reflect ageing-related impairments in neural activity patterns.

## Discussion

Using high spatiotemporal resolution MEG imaging, this study determined the detailed characteristics of band-limited spectral power and spectral aperiodic slope in ageing. Our study elaborated the subtle changes of neural power spectrum within the sixth to ninth decade of life. In a subset of individuals uniformly assessed using a cognitive battery, we also examined the relationships between neurophysiological changes and cognitive abilities. Specifically, we examined whether a given spectral change was either positively or negatively associated with better cognition, to determine their role either as compensatory or compromised, respectively, in the ageing brain. We found that ageing-related frontal predominant delta–theta reductions and inferolateral–temporal alpha increases show positive associations with better cognition, thus representing likely compensatory neural mechanisms supporting cognitive resilience in ageing. In contrast, beta power reductions and aperiodic slope reductions showed negative associations with cognition and represented likely compromised neural mechanisms in ageing. Understanding the specific neurophysiological indices that support cognitive ageing may provide crucial information to determine how these become vulnerable in ageing as well as in neurodegenerative diseases like Alzheimer’s disease, where age is the highest risk factor.

### Spectral changes during older age are distinct from lifespan spectral trajectories

The lifespan trajectory of alpha power is well documented as an increase from early childhood up to puberty and a decrease thereon.^3^ This generalized trajectory may seem contradictory to our finding from the linear fit analysis showing an overall increase in alpha PSD with ageing in later years. However, it is important to consider that the downward lifespan trajectory of alpha is driven by the power drop from the puberty peak when compared with the tenth decade. This generalized trajectory masks the subtle changes in later decades of life. Indeed, investigations that categorically examined alpha power during the sixth to tenth decades reported either stable alpha power in,^7,8^ or slightly increased alpha,^30^ as well as maintenance of alpha peak above 9Hz.^9,30-32^ Collectively, these observations argue against a consistent reduction of alpha with ageing and indicate that alpha power possibly gains stability and strength with ageing, especially in the absence of neurological diseases. Our findings are not only consistent with such phenomenon but also demonstrate the detailed region-specific spatiotemporal mapping of increased alpha in healthy ageing.

Unlike alpha, low-frequency delta and theta and higher-frequency beta do not show consistent rhythmic oscillatory peaks in the resting human brain.^5^ The band-limited spectral power without a clear oscillatory peak can represent periodic neural activity with a range of central frequencies or simply the weighted sum of varying frequency, amplitude and phase, regardless of signal periodicity. Notwithstanding such ambiguity, delta–theta and beta power is still useful to infer changes across brain states and traits. The low-frequency delta and theta power in older healthy adults has been more consistently reported as being reduced when compared with younger participants.^33-35^ EEG and MEG studies showing lifespan trajectories of delta–theta have demonstrated a downward slope from young adulthood (18 years) to elderly (85 years).^15,36^ A population study using MEG to examine spectral power within every decade of life showed that delta and theta power reduces after 50 years.^8^ Another population study using EEG showed delta–theta power to be mostly stable or reduced during the adulthood.^7^ Consistent with these reports, in our linear fitting analyses, we found an overall reduction of delta–theta power with ageing which we further demonstrated to be of a frontal predominant spatial distribution. Other reports, however, have shown increased low-frequency power in older subjects.^37,38^ It is noteworthy though the possibility of increased delta–theta power may result from confounded study samples including elderly participants with subclinical neurodegenerative processes. For example, studies that specifically examined EEG features in individuals with and without neurological conditions as well as those that longitudinally followed successfully aged individuals indicate that delta–theta power does not increase with age in the context of good health status.^39,40^ Beta spectral power has been reported with a range of methodological differences related to band cut-offs. A large EEG population study showed that the lifespan trajectory of 13–20 Hz band power has a negative slope, whereas that of 20–30 Hz has an inverted ‘U’ shape where it increases until age 25 years and then start to decrease.^7^ This collective result is consistent with our finding of reduced beta power within 13–30 Hz band during the 55–90 age range as we further demonstrate the spatial pattern of such power reduction.

### Alpha and delta–theta power changes with normative ageing support better cognition, while beta power and aperiodic slope changes do not

It is well documented that increased alpha power but reduced delta–theta power in the resting brain are associated with good cognitive performance in multiple domains. For example, higher alpha power and peak frequency are associated with greater processing speed,^41^ attention and memory.^42^ A study including subjects ranging from young to older adults demonstrated that, when age is regressed out, higher alpha and reduced theta power correlated with greater visual short-term memory.^43^ In a study of healthy elderly, better working memory correlated with lower theta power at rest.^44^ Our findings of above-median subgroup having lower delta–theta and higher alpha are consistent with these frequency-specific relationships, and the PLSR analyses illustrated the regional patterns of these neurobehavioural correlations. A key finding from our study is that the directional changes in delta–theta and alpha oscillatory activity that were observed with ageing are the same directional changes associated with better cognition, which included reductions in delta–theta and increases in alpha. This suggests that the spectral changes in delta–theta and alpha oscillations seen in normal ageing may serve as compensatory neural mechanisms and are likely to play a role in supporting cognitive resilience in the ageing process. In contrast to alpha and delta–theta, the directional changes of beta and aperiodic slope in ageing were inverse of what was associated with better cognitive scores. For example, beta power and slope both decreased with ageing, whereas it was higher beta power and higher slope values that were linked to better cognitive performance. This inverse relationship indicates that reductions in beta power and the aperiodic slope may reflect compromised neural mechanisms in the ageing brain. It would be highly informative in future investigations to examine the synaptic and proteomic measures associated with these spectral features.

### Greater network hyperexcitability is indexed by aperiodic slope reductions in ageing

The aperiodic spectral exponent (‘1/f slope’) has emerged as a promising candidate to represent the excitatory–inhibitory balance (*E*/*I*). Converging evidence has linked aperiodic slope changes to network hyperexcitability in epilepsy^19,45^ as well as in other diseases where *E*/*I* balance is considered abnormal including Alzheimer’s disease,^46^ autism^47^ and attention-deficit hyperactivity disorder.^48^ Our results from the linear model fitting analysis showing reduced spectral aperiodic slope with advancing age are consistent with previous reports comparing older adults to young, suggesting greater network hyperexcitability associated with ageing.^17^ The novel finding from this study is to show that such network hyperexcitability is localized to frontal lobes—an area where predominant neuronal loss happens with ageing. Our study, however, showed mixed results on the associations between aperiodic slope and cognition. For example, our median split analysis showed that below-median subgroup has significantly higher aperiodic slopes depicting greater network hyperexcitability than above-median subgroup, while the PLSR only showed a weak association of aperiodic slope and cognitive scores. These results suggest the possibility of hyperexcitability having more globally mediated effect than regional, with additional mediators between altered *E*/*I* and cognitive outcomes.^49^

### Compensatory and compromised spectral changes in ageing have important implications for Alzheimer’s disease research

The characteristic spectral change in Alzheimer’s disease includes increased delta–theta and reduced alpha spectral power.^4^ What we observe in healthy ageing here is in sharp contrast to this: a decrease in delta–theta and increase in alpha power. Therefore, alterations of neural oscillatory activity in Alzheimer’s disease can be detected with high sensitivity from healthy ageing. Indeed, increased delta–theta and reduced alpha electrophysiological measures have been demonstrated to reliably indicate Alzheimer’s disease-related neurophysiological abnormalities.^4,22,23,50,51^ Furthermore, our results suggest that it is critical for future neurobehavioural studies to covary the effects of age. For example, it is unclear whether the higher delta–theta associations with better cognition are driven by younger study participants in a given cohort study.^52^ Likewise, the positive relationship between ageing and aperiodic slope reductions suggests that when quantifying *E*/*I* in clinical populations, it is important to covary the effect of age. A better characterization of age-related changes in neural oscillatory networks will deliver more accurate estimates of Alzheimer’s disease-related pathological changes that can be used as important functional biomarkers in Alzheimer’s disease clinical trials.

### Limitations

Our study is not without limitations. First, our sample size of *n* = 40 with uniform data collection across a comprehensive standard neuropsychological battery is lower than ‘population’ studies reporting psychological data. However, we would like to emphasize that the findings are a valuable addition to the current ageing knowledgebase given the high resources demands to collect high spatiotemporal resolution source reconstructed MEG imaging with comprehensive cognitive assessments in a well-characterized healthy elderly cohort. Second, as for any cross-sectional ageing study, the effect of sampling to disease susceptibility is an important consideration. Sampling can lead to an underestimation of age-related changes as elderly people with more health problems are less likely to survive. Cross-sectional analyses usually confound averages as well as within- and between-subject variations, although our full sample is large enough to make reliable conclusions beyond these confounds. Third, the current study findings of PSD used specific frequency bands based on most commonly used cut-offs, including a combined delta–theta, alpha and beta bands. These conventional boundaries, however, are not clearly defined on biological underpinnings, and it is likely the subcomponents of these bands having specific correlations to physiological processes than the full-band activity. Future studies with larger study populations can be utilized to delineate these finer frequency-specific changes with ageing. Finally, our analyses including cognitive tests included cognitive test performance within 7.85 ± 5.2 months from the MEG scan. While a 3-month time window is optimal to make clinical conclusions about cognitive performance from the standard bedside testing, we believe that our data set that is exclusively from cognitively unimpaired individuals is minimally affected by a time difference of 7.85 months. Furthermore, all participants in this study were followed up with yearly cognitive evaluations for many years and we can exclude underlying neurodegenerative diseases with high clinical confidence.

## Supplementary Material

fcaf131_Supplementary_Data

## Data Availability

All data associated with this study are presented in the paper. Anonymized subject data will be shared on request from qualified investigators for the purposes of replicating procedures and results and for other non-commercial research purposes within the limits of participants’ consent. Correspondence and material requests should be addressed to Kamalini.ranasinghe@ucsf.edu or Srikantan.nagarajan@ucsf.edu.
