# Supplementary material for: Neurophysiological signatures of ageing: compensatory and compromised neural mechanisms
Source: Brain Commun. 2025 Apr 4;7(2):fcaf131. doi: 10.1093/braincomms/fcaf131 (PMC12006661; doi:10.1093/braincomms/fcaf131)
Supplement: fcaf131_Supplementary_Data [file fcaf131_supplementary_data.pdf]

**SUPPLEMENT**

## 1. Supplementary Methods:

### 1.1. Neuropsychological Assessment

A standard battery of neuropsychological tests were used to assess major cognitive domains and n=40 participants were evaluated with a uniform testing procedure to document executive, memory and processing speed ability[1, 2]. Executive function tests included modified trail-making, design fluency, and letter fluency (D words) tests. The modified trail-making test required the participant to serially alternate between numbers and days of the week, evaluating set-shifting and sequencing within a required time window of 2-minutes and the dependent measure was calculated as the number of correct connections made per minute. Design fluency was measured using the filled dots condition from the design fluency subscale of the Delis-Kaplan Executive Function Scale (DKEFS)[3] and was scored as the number of correct designs generated within a minute. Memory function tests included California Verbal Learning Test (CVLT 16 item)[4] short and long recall and modified-Rey recall. The latter included drawing the Benson figure from memory after a 10-minute delay and was scored on a 17-point scale[5]. Processing speed was assessed via Stroop Color Naming and cognitive control via the Stroop Inhibition test. [6]. Each individuals' component test scores from executive (modified trails, design fluency, and letter fluency) and memory (CVLT short and long recall and modified-Ray recall) domains were converted into test-specific z-scores, based on age- education- and sex-matched normative datasets from the UCSF-MAC, and then averaged to create domain specific scores (for each individual).

**2. Supplementary figure 1:**

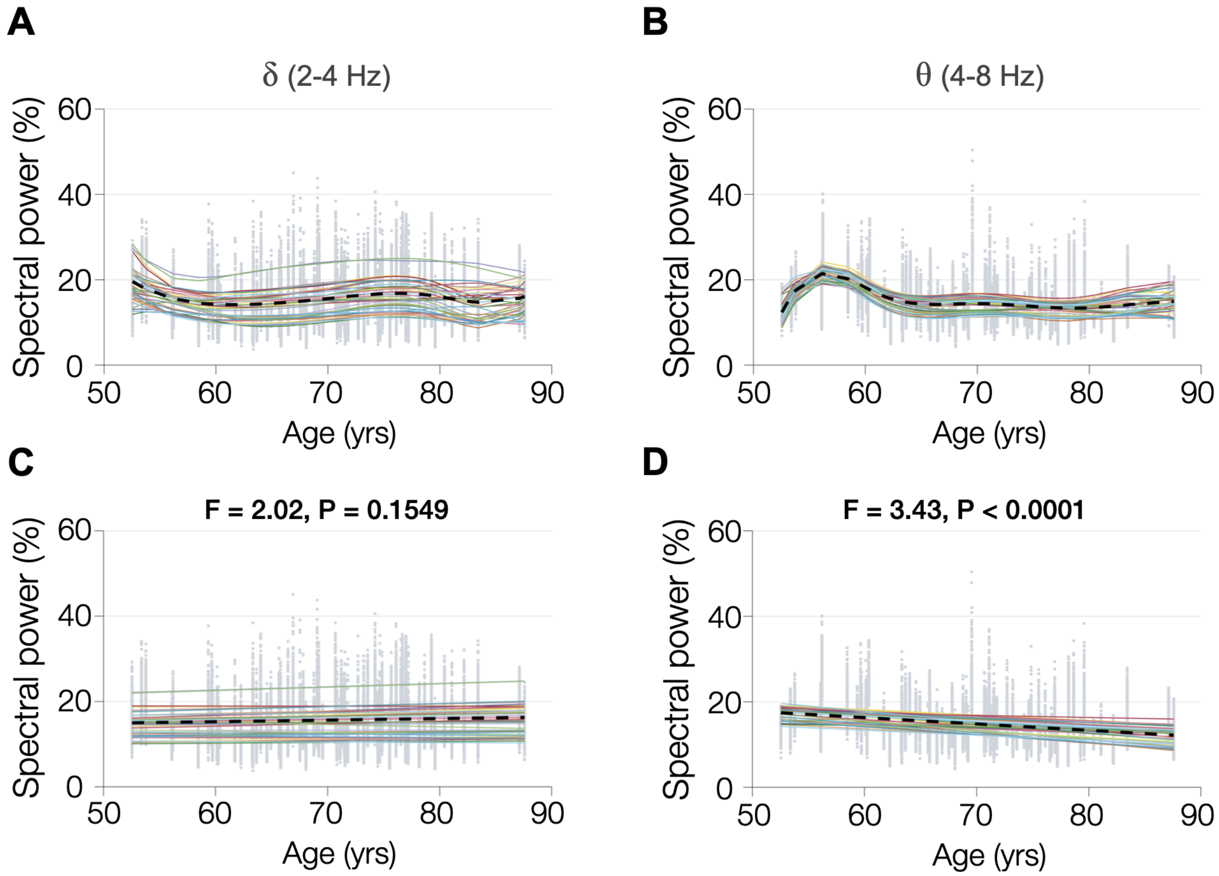


**Spectral changes with aging in delta and theta bands.** Band-limited spectral-power within delta (2-4 Hz), and theta (4-8 Hz) frequency bands is examined in the full cohort (n=70). Spline fits for region-specific spectral data (Brainnetome atlas modules) showed the trajectory of change across age for each band-limited spectral-power (A-B). Linear model fits showed that delta band activity does not show a statistically significant change with aging (C) while a theta band showed a low-grade decrease with aging (D) (K). Colored lines represent the spectral measures in each of the 48 modular level parcellations defined in the Brainnetome atlas and dotted line represent the average across all regions. Abbreviations: SP, spectral-power

**3. Supplementary figure 2:**

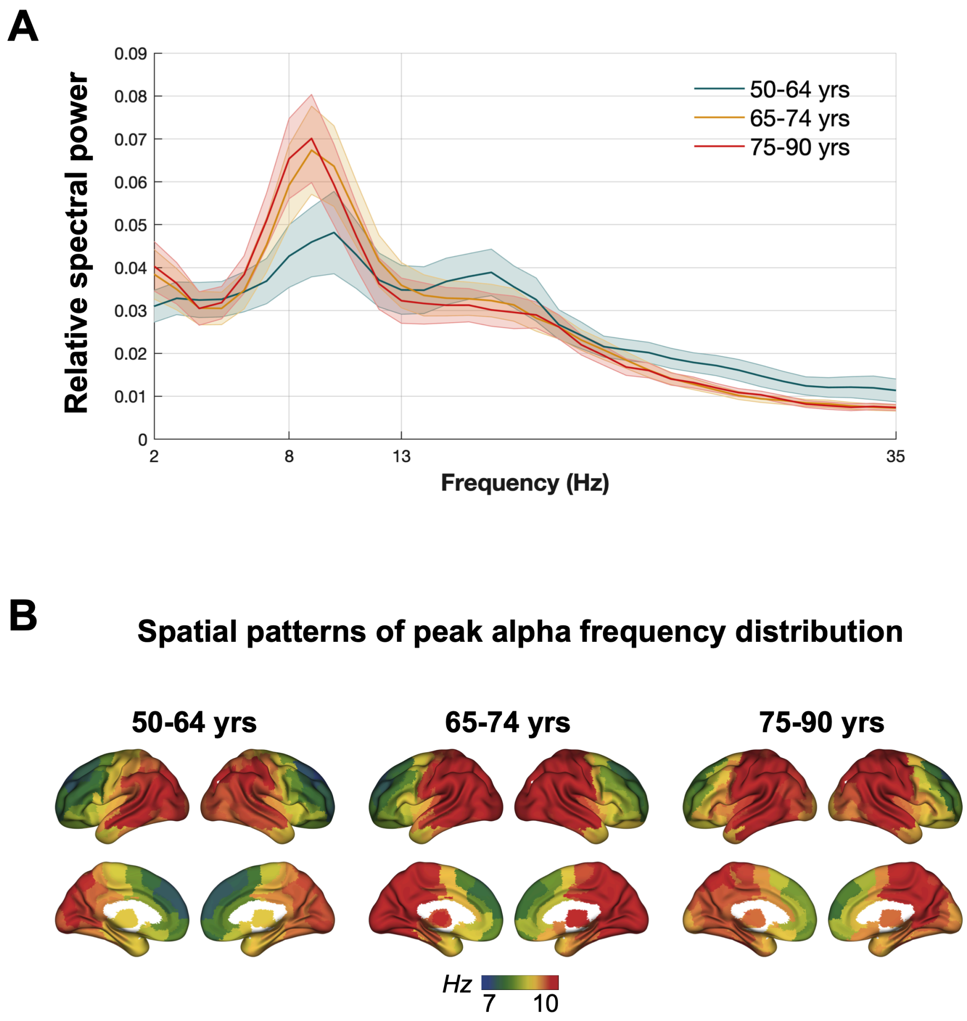


**Power spectra and spatial patterns of alpha peak frequency changes with aging.** Spectral power density computed as relative power for the full frequency distribution of 2-35 Hz band is shown in subplot A for each tercile bin of the age distribution in the full cohort, which included the age bins of 50-64 (n=21), 65-74 (n=29), and 75-90 (n=20). In subplot B the brain renderings depict the peak alpha frequency (8 – 12 Hz band) in a regional analysis for the cortical regions defined in the Brainnetome atlas in each cohort.

**4. Supplementary Table 1: Demographic characteristics and cognitive test performance of participants in the full cohort vs. sub cohort used for PLS analysis**

| **Variable** | **Full cohort** (n=70) | | **Sub-cohort with cognitive testing** (n=40) | | ***t (P value)*** |
|---|---|---|---|---|---|
| | **Mean (SD)** | **Range (min – max)** | **Mean (SD)** | **Range (min – max)** | |
| Age (years) | 69.8 ± 8.21 | 52.54 - 87.58 | 72.47 ± 7.39 | 53.38 - 87.58 | -1.75 (0.0829) |
| Education (years) | 17.43 ± 1.97 | 12 - 20 | 17.83 ± 1.95 | 14 - 20 | -1 (0.3179) |
| MMSE | 29.43 ± 0.91 | 25 - 30 | 29.48 ± 0.99 | 25 - 30 | -0.24 (0.8078) |
| CDR | 0.01 ± 0.08 | 0 - 0.5 | 0.01 ± 0.08 | 0 - 0.5 | 0.11 (0.9115) |
| CDRBOX | 0.03 ± 0.12 | 0 - 0.5 | 0.03 ± 0.11 | 0 - 0.5 | 0.16 (0.8734) |
| Modified trails | 0.65 ± 0.25 | 0.2 - 1.17 | 0.59 ± 0.23 | 0.2 - 1.08 | 1.04 (0.3039) |
| Design fluency | 12.82 ± 3.56 | 6 - 20 | 12.68 ± 3.72 | 6 - 20 | 0.18 (0.8596) |
| Modified Rey copy | 15.5 ± 0.77 | 14 - 17 | 15.56 ± 0.76 | 14 - 17 | -0.36 (0.7213) |
| Modified Rey recall | 12.12 ± 2.79 | 5 - 16 | 12.25 ± 2.83 | 5 - 16 | -0.2 (0.8425) |
| Repetition | 4.83 ± 0.42 | 3 - 5 | 4.85 ± 0.36 | 4 - 5 | -0.23 (0.8169) |
| Digit span forwards | 7.16 ± 1.34 | 4 - 9 | 7.06 ± 1.41 | 4 - 9 | 0.35 (0.7301) |
| Digit span backwards | 5.75 ± 1.32 | 3 - 8 | 5.76 ± 1.6 | 3 - 8 | -0.06 (0.9532) |
| Phonemic fluency | 17.52 ± 5.4 | 8 - 33 | 17.66 ± 6.02 | 8 - 33 | -0.1 (0.9186) |
| Category fluency | 23.93 ± 4.88 | 13 - 39 | 24.33 ± 4.6 | 16 - 39 | -0.4 (0.6887) |
| Processing speed | 86.72 ± 15.51 | 46 - 133 | 84.85 ± 16.78 | 46 - 133 | 0.52 (0.6043) |
| Stroop inhibition | 52.06 ± 14.07 | 1 - 92 | 50.27 ± 15.53 | 1 - 92 | 0.54 (0.5942) |
| VOSP number location | 9.13 ± 1.23 | 5 - 10 | 9.24 ± 1.16 | 5 - 10 | -0.41 (0.685) |
| GDS | 2.62 ± 2.58 | 0 - 11 | 2.69 ± 2.46 | 0 - 11 | -0.13 (0.8983) |
| CVLT learning | 53.05 ± 10.67 | 32 - 71 | 53.58 ± 10.81 | 33 - 71 | -0.25 (0.8066) |
| CVLT short delay recall | 11.92 ± 2.86 | 5 - 16 | 12.33 ± 2.67 | 6 - 16 | -0.73 (0.4684) |
| CVLT long delay recall | 12.45 ± 2.89 | 5 - 16 | 12.78 ± 2.51 | 7 - 16 | -0.6 (0.5487) |

Values for all variables except for sex handedness and race are means ±SD.

*P values are reported from a t-test for age, education, and all the cognitive tests, and from Pearson $\chi 2$ test for sex.

## REFERENCES


[1] Ranasinghe KG, Rankin KP, Lobach IV, Kramer JH, Sturm VE, Bettcher BM, et al. Cognition and neuropsychiatry in behavioral variant frontotemporal dementia by disease stage. Neurology. 2016;86:600-10.

[2] Kramer JH, Jurik J, Sha SJ, Rankin KP, Rosen HJ, Johnson JK, et al. Distinctive neuropsychological patterns in frontotemporal dementia, semantic dementia, and Alzheimer disease. Cogn Behav Neurol. 2003;16:211-8.

[3] Delis DC, Kaplan E, Kramer JH. Delis-Kaplan Executive Function System. San Antonio, TX: The Psychological Corporation; 2001.

[4] Delis DC, Kramer JH, Kaplan E, Ober BA. California Verbal Learning Test - Second Edition, Adult Version. San Antonio, TX: The Psychological Corporation; 2000.

[5] Possin KL, Laluz VR, Alcantar OZ, Miller BL, Kramer JH. Distinct neuroanatomical substrates and cognitive mechanisms of figure copy performance in Alzheimer's disease and behavioral variant frontotemporal dementia. Neuropsychologia. 2011;49:43-8.

[6] Golden CJ. Stroop Color and Word Test: Revised examiner's manual. Wood Dale, IL: Stoelting Co; 2002.
